# 

**Published:** 1882-07

**Authors:** 


					﻿Whoi|e Number,
jCCCCL.
July, 1882.
Number 1,'
Vol. XLV.
THE
CHICAGO
MEDICAL
T ournal and Examiner
EDITORS:
N. S. DAVIS, M.D., LL.D. JAS. NEVINS HYDE, A.M.. M.D,
DANIEL R. BROWER M.D.
CHICAGO:
PUBLISHED BY THE CHICAGO MEDICAL PRESS ASSOCIATION,
188 Clark Street.
^Read the Notice of this Journal among Advertisements.
				

